# Serum CD93 as a Potential Diagnostic Biomarker for Endometrial Cancer: A Case–Control Study

**DOI:** 10.3390/jcm15093409

**Published:** 2026-04-29

**Authors:** İsmail Bağlar, Fatih Şanlıkan, Esra Keles, Sahra Sultan Kara, Cansu Ergenç Özdaş, Yeliz Çeçen Dönmez, Hafize Uzun

**Affiliations:** 1 Department of Obstetrics and Gynecology, University of Health Sciences, Kartal Dr. Lütfi Kırdar City Hospital, 34865 Istanbul, Türkiye; ismailbg@gmail.com (İ.B.); sahracavusoglu@gmail.com (S.S.K.); yelizcecendonmez@gmail.com (Y.Ç.D.); 2 Department of Gynecologic Oncology, University of Health Sciences, Kartal Dr. Lütfi Kırdar City Hospital, 34865 Istanbul, Türkiye; fatihsanlikan1@gmail.com (F.Ş.); dresrakeles@gmail.com (E.K.); 3 Management Department, Business School, Ankara Yildirim Beyazit University, 06010 Ankara, Türkiye; cansuergenc@aybu.edu.tr; 4 Department of Biochemistry, Faculty of Medicine, Istanbul Atlas University, 34408 Istanbul, Türkiye

**Keywords:** endometrial cancer, CD93, circulating biomarker, angiogenesis, diagnostic accuracy, ectodomain shedding

## Abstract

**Objectives:** CD93, an angiogenesis-related transmembrane glycoprotein, is transcriptomically downregulated in uterine corpus endometrial carcinoma, yet circulating protein levels have not been clinically evaluated. This study aimed to evaluate serum CD93 as a diagnostic biomarker for EC and to examine its association with clinicopathological parameters. **Methods:** In this single-center case–control study, serum CD93 concentrations were measured by enzyme-linked immunosorbent assay in 46 patients with histologically confirmed primary EC and 35 controls with histologically verified benign gynecological pathology. Logistic regression and receiver operating characteristic (ROC) curve analyses were performed. **Results:** Serum CD93 was significantly lower in EC patients than controls (median 4.55 [IQR 3.51–6.97] vs. 10.24 [7.18–12.14] ng/mL; *p* < 0.001). In multivariable analysis adjusted for age and body mass index, lower CD93 remained independently associated with EC (OR = 0.521; 95% CI 0.061–0.720; *p* < 0.001). ROC analysis yielded an area under the curve of 0.845 (95% CI 0.759–0.921), with 82.6% sensitivity and 74.3% specificity at a cut-off of 7.338 ng/mL. CD93 levels showed no significant association with histological subtype, grade, lymphovascular space invasion, nodal metastasis, or recurrence. **Conclusions:** Serum CD93 is significantly reduced in EC and demonstrates independent diagnostic performance, supporting its prospective validation as a non-invasive biomarker in larger multicenter cohorts.

## 1. Introduction

Endometrial cancer (EC) is the most prevalent gynecological malignancy in developed nations. According to GLOBOCAN 2022 estimates, endometrial cancer accounted for approximately 420,242 new cases and 97,704 deaths globally, making it the sixth most common cancer in women [1]. The International Agency for Research on Cancer (IARC) projects a substantial escalation in EC incidence over the coming decades, with an estimated 606,798 new cases anticipated by 2040 and approximately 694,000 cases projected by 2045, representing a 63% increase from 2022 figures [1,2]. Its incidence has steadily increased over recent decades, driven largely by the escalating rates of obesity, metabolic syndrome, population aging and lifestyle changes—established risk factors for type I (endometrioid) EC [2,3]. Most ECs are diagnosed at an early stage, typically following the onset of postmenopausal bleeding, and are associated with favorable prognosis; however, high-grade histological subtypes, including serous carcinoma, clear cell carcinoma, and carcinosarcoma frequently present with aggressive clinical behavior, advanced disease, and poor survival outcomes [3,4]. Current diagnostic strategies rely predominantly on invasive procedures such as endometrial biopsy and dilatation and curettage, which, although effective, may cause patient discomfort, carry procedural risks, and are subject to sampling error [4,5]. These limitations underscore an unmet clinical need for reliable, non-invasive circulating biomarkers that could facilitate early detection, risk stratification, and triage of patients with suspected endometrial malignancy.

Conventional serum biomarkers have been investigated in EC but have demonstrated limited sensitivity and specificity, particularly for early-stage disease [5,6]. Recent advances in genomic, transcriptomic, and proteomic profiling have expanded the landscape of candidate biomarkers; yet, few have achieved the clinical validation necessary for integration into routine practice [6,7]. Compared with existing circulating markers such as CA-125, which yields area under the curve (AUC) values typically between 0.60 and 0.75 for distinguishing EC from benign conditions, and HE4, whose performance has been inconsistent across populations, there remains a compelling rationale for identifying novel serum-based markers with superior discriminative capacity [5,6,8].

CD93 (also known as C1qRp or AA4.1) is a type I transmembrane glycoprotein belonging to the group XIV C-type lectin-like domain (CTLD) superfamily [9]. It is predominantly expressed on vascular endothelial cells, where it plays a critical role in angiogenesis, endothelial cell adhesion, migration, and maintenance of the vascular barrier [9,10]. Structurally, the extracellular domain of CD93 interacts with key ligands, including multimerin-2 (MMRN2) and insulin-like growth factor-binding protein 7 (IGFBP7), both of which modulate endothelial function within the tumor microenvironment (TME) [11,12]. Under physiological conditions, CD93 contributes to vascular integrity. The extracellular domain of CD93 is subject to proteolytic cleavage (ectodomain shedding) mediated by matrix metalloproteinases (MMPs) particularly MMP-2 and members of the ADAM family of sheddases on the surface of vascular endothelial cells and activated myeloid cells, including monocytes and neutrophils [13]. This shedding releases a soluble form (sCD93) into the circulation that retains its C-type lectin-like domain and EGF-like repeats, enabling continued interaction with integrins and facilitating efferocytosis—the phagocytic clearance of apoptotic cells [13,14]. Following ectodomain shedding, the intracellular domain (ICD) of CD93 undergoes further cleavage and may translocate to the nucleus, where it participates in transcriptional regulation. The primary intracellular signaling mechanism described for CD93 involves the CD93/Cbl/Crk adaptor protein pathway, which regulates Rho family GTPase activity and thereby governs cytoskeletal dynamics, migration, and adhesion in endothelial cells [10]. Serum sCD93 has been proposed as a biomarker in inflammatory and neoplastic diseases [13,15].

In several solid malignancies, CD93 expression in tumor-associated vasculature is markedly upregulated and correlates with adverse clinical outcomes. Elevated vascular CD93 has been reported in glioblastoma [16,17], gastric cancer [18], hepatocellular carcinoma [19], and colorectal cancer [20], where it promotes β1 integrin activation, fibronectin fibrillogenesis, and vascular abnormalization [11,16]. In these contexts, CD93 upregulation is typically driven by vascular endothelial growth factor (VEGF) signaling in the TME [17,21], and pharmacological blockade of the CD93 pathway has been shown to normalize tumor vasculature, enhance drug delivery, and improve responsiveness to immunotherapy [21].

However, emerging evidence from pan-cancer bioinformatics analyses has revealed that CD93 expression is not uniformly upregulated across all tumor types. Two independent transcriptomic studies demonstrated that CD93 is significantly downregulated in uterine corpus endometrial carcinoma (UCEC) and kidney renal papillary cell carcinoma (KIRP), in contrast to its upregulation in most other solid tumors [22,23]. These findings suggest that CD93 may exert organ-specific functions that differ fundamentally between tumor microenvironments. Despite these computational observations, very few clinical studies to date have measured circulating CD93 protein levels in patients with EC.

Although previous transcriptomic studies have suggested a downregulation of CD93 in uterine corpus endometrial carcinoma tissues, to the best of our knowledge, no clinical study to date has evaluated the circulating serum levels of CD93 in patients with EC. The present study was designed to quantify serum CD93 levels in patients with histologically confirmed EC compared with controls with benign gynecological conditions and to evaluate the diagnostic utility of circulating CD93 as a non-invasive biomarker. Furthermore, we sought to investigate, for the first time, the association between serum CD93 levels and clinicopathological characteristics, including histological subtype, tumor grade, myometrial invasion, lymphovascular space invasion (LVSI), lymph node metastasis, and disease recurrence.

## 2. Materials and Methods

### 2.1. Study Design and Population

This single-center, case–control study was conducted at the Department of Obstetrics and Gynecology of a tertiary hospital. The case group comprised 46 consecutive patients with histologically confirmed primary EC who underwent comprehensive surgical staging between April 2024–December 2025. Surgical staging included total hysterectomy, bilateral salpingo-oophorectomy, and systematic pelvic and para-aortic lymphadenectomy in accordance with the 2023 International Federation of Gynecology and Obstetrics (FIGO) staging system [4]. Histological classification and tumor grading were performed by two experienced gynecological pathologists according to World Health Organization (WHO) criteria.

The control group consisted of 35 women who underwent total abdominal hysterectomy for benign indications (uterine leiomyomata, adenomyosis, or benign endometrial polyps) during the same study period, with benign pathology confirmed on definitive histopathological examination.

Inclusion criteria for EC cases were: (i) age ≥ 18 years; (ii) histologically confirmed primary EC on surgical specimen; and (iii) availability of preoperative serum samples obtained prior to surgical intervention. Exclusion criteria applied to both groups were: (i) concurrent or prior malignancy; (ii) active systemic infection within 4 weeks of enrollment; (iii) documented autoimmune or chronic inflammatory disease requiring immunosuppressive therapy; (iv) receipt of neoadjuvant chemotherapy, radiotherapy, or hormonal therapy prior to blood sampling; and (v) insufficient serum volume for duplicate biomarker analysis.

The study protocol was approved by the Research Ethics Committee of the University of Health Sciences, Kartal Koşuyolu Training and Research Hospital (Approval Number: 2026/06/1425, Date: 24 March 2024) and conducted in accordance with the Declaration of Helsinki. All participants provided written informed consent prior to enrollment.

### 2.2. Sample Collection and Biomarker Quantification

Peripheral venous blood (10 mL) was collected by antecubital venipuncture from all participants during preoperative evaluation. Following collection, specimens were maintained in an upright position and allowed to clot at room temperature (20–25 °C) for 30 min. Tubes were then centrifuged at 3000× *g* for 10 min at 4 °C. The resulting serum supernatant was carefully aspirated, avoiding disturbance of the buffy coat layer, and distributed into multiple 500 μL aliquots in sterile, DNase/RNase-free polypropylene cryovials. Aliquots were snap-frozen and stored at −80 °C in a temperature-monitored biorepository. The interval between venipuncture and cryopreservation did not exceed 2 h for any specimen. All samples were thawed only once for batch analysis, thereby eliminating freeze-thaw-related protein degradation as a source of analytical bias. All samples were analyzed in duplicate, with an intra-assay CV < 10%, inter-assay CV < 12%, and standard curve range of 0.5–20 ng/mL.

### 2.3. Assessment of Serum CD93

Serum CD93 concentrations were quantified using a commercially available sandwich ELISA kit for human soluble CD93 (sCD93) (CSB-EL004960HU; Cusabio Biotech Co., Wuhan, China) in accordance with the manufacturer’s protocol. Briefly, serum samples (1:5 dilution) and recombinant standards were incubated in duplicate on microplates pre-coated with a monoclonal anti-CD93 antibody (2 h, 37 °C). Following washing, a biotinylated detection antibody and streptavidin–HRP were sequentially applied, and color development was achieved using TMB substrate. Absorbance was read at 450 nm with 570 nm correction using a calibrated microplate reader. A seven-point standard curve was generated using serial dilutions and fitted with a four-parameter logistic (4-PL) model. Sample concentrations were calculated from mean duplicate OD values. The assay’s analytical performance included a minimum detectable dose of 0.12 ng/mL, with intra- and inter-assay CVs of <8% and <12%, respectively [12,13]. Quality control sera (low, medium, high) were included on each plate, and runs were accepted only if QC values were within ±15% of target concentrations. Samples exceeding the calibration range were reanalyzed after appropriate dilution.

### 2.4. Conventional Tumor Marker Assessment

Serum CA-125 and CA19-9 concentrations were determined in the EC group as part of routine preoperative clinical evaluation using chemiluminescent microparticle immunoassay (CMIA) on an automated platform (Architect i2000SR; Abbott Diagnostics, Abbott Park, IL, USA). Results were extracted from institutional laboratory records. These markers were not systematically measured in control subjects; consequently, between-group comparative analyses for CA-125 and CA19-9 were not undertaken.

### 2.5. Clinical and Pathological Data Collection

Baseline demographic data, including age, body mass index (BMI), parity, and menopausal status, were recorded for all participants. For the EC group, detailed clinicopathological information was extracted from surgical and pathological reports, including histological type (endometrioid, serous, clear cell, or carcinosarcoma), tumor grade (grades 1–3), tumor size, depth of myometrial invasion (<1/2 or ≥1/2), presence or absence of LVSI, pelvic and para-aortic lymph node metastasis, and disease recurrence status during follow-up.

### 2.6. Statistical Analysis

Statistical analyses were performed using SPSS (version 26.0; IBM Corp., Armonk, NY, USA). Continuous variables were expressed as median with interquartile range (IQR) and compared between groups using the Mann–Whitney U test, given the non-normal distribution of the data. Categorical variables were presented as frequencies with percentages and compared using the chi-square test or Fisher’s exact test, as appropriate. The Kruskal–Wallis test was used for comparisons among three or more groups.

Sample size estimation: The sample size was estimated a priori to detect a statistically significant AUC for serum CD93 in discriminating EC from controls. Assuming an expected AUC of 0.80, a null hypothesis AUC of 0.50, a two-sided alpha of 0.05, a statistical power of 80%, and an anticipated case-to-control ratio of approximately 1.3:1, a minimum of 38 cases and 30 controls were required based on the methodology of Hanley and McNeil [24]. The final sample of 46 EC patients and 35 controls exceeds these requirements and provides adequate statistical power for the primary diagnostic performance analysis. Post hoc power analysis confirmed that the study achieves approximately 95% power to detect the observed AUC of 0.845 at a two-sided significance level of 0.05; however, the sample is insufficient for adequately powered subgroup analyses within clinicopathological strata.

Univariable and multivariable logistic regression analyses were conducted to evaluate the association between clinical variables (age, BMI, and serum CD93) and EC status. L2-regularized logistic regression was applied using the scikit-learn framework (regularization parameter C = 1.0), and bootstrap-based 95% confidence intervals (CIs) were estimated from 1000 iterations. Menopausal status was not included as a separate covariate due to its strong collinearity with age; adjustment for age as a continuous variable captures the variance attributable to menopausal transition without introducing multicollinearity. A sensitivity analysis confirmed that inclusion of parity and menopausal status as separate covariates did not materially alter the point estimates or statistical significance of the CD93 association. The diagnostic performance of serum CD93 was assessed using receiver operating characteristic (ROC) curve analysis, with calculation of the area under the curve (AUC), sensitivity, specificity, positive predictive value (PPV), and negative predictive value (NPV). The optimal cut-off value was determined using the Youden index. A two-sided *p*-value < 0.05 was considered statistically significant for all analyses.

## 3. Results

### 3.1. Baseline Characteristics

The baseline demographic and clinical characteristics of the study population are presented in Table 1. Patients with EC were significantly older than control subjects (median age: 63.00 [IQR 55.75–69.00] vs. 45.00 [IQR 32.50–55.00] years; *p* < 0.001). BMI was also significantly higher in the EC group compared with the control group (median: 37.00 [IQR 30.00–43.00] vs. 28.00 [IQR 27.00–29.00] kg/m^2^; *p* < 0.001). Parity did not differ significantly between groups (*p* = 0.377). Menopausal status distribution differed significantly between the two groups (*p* < 0.001): 40 of 45 EC patients (88.9%) were postmenopausal compared with 14 of 35 (40.0%) in the control group.

### 3.2. Serum Biomarker Levels

Serum CD93 levels are summarized in Table 2. The median serum CD93 concentration was significantly lower in the EC group than in the control group (4.55 [IQR 3.51–6.97] vs. 10.24 [IQR 7.18–12.14] ng/mL; *p* < 0.001). In the EC group, the median CA-125 level was 15.9 [IQR 7.6–26.8] U/mL (available for 42 patients), and the median CA19-9 level was 13.0 [IQR 6.9–19.0] U/mL (available for 41 patients).

### 3.3. Association Between Serum CD93 Levels and Clinicopathological Parameters

The associations between serum CD93 levels and clinicopathological characteristics in the EC group are presented in Table 3. Serum CD93 levels did not differ significantly according to menopausal status (*p* = 0.396), histological type (*p* = 0.706), tumor grade (*p* = 0.722), tumor size categorized as ≤4 cm vs. >4 cm (*p* = 0.506), LVSI (*p* = 0.275), pelvic lymph node involvement (*p* = 1.000), para-aortic lymph node involvement (*p* = 0.457), or recurrence (*p* = 0.218). A borderline association was observed between serum CD93 levels and depth of myometrial invasion (*p* = 0.093): patients with deep myometrial invasion (≥1/2) had lower median CD93 levels (3.52 [IQR 2.82–4.71] ng/mL) compared with those with superficial invasion (<1/2; 4.68 [IQR 3.57–6.83] ng/mL).

Regarding the distribution of clinicopathological features across histological subtypes, endometrioid carcinoma was the predominant subtype (34 of 46, 73.9%), followed by serous carcinoma (4 of 46, 8.7%), clear cell carcinoma (2 of 46, 4.3%), and carcinosarcoma (1 of 46, 2.2%). Among endometrioid carcinomas, 16 were grade 1, 11 were grade 2, and 7 were grade 3; the 4 serous carcinomas were uniformly high-grade, and both clear cell carcinomas and the sole carcinosarcoma were classified as high-grade lesions. Deep myometrial invasion (≥1/2) was observed in 8 of 34 endometrioid, 2 of 4 serous, and 1 of 2 clear cell carcinomas. Lymph node metastasis was identified in 2 endometrioid, 1 serous, and 1 clear cell carcinoma.

### 3.4. Logistic Regression Analysis

The results of univariable and multivariable logistic regression analyses are presented in Table 4. In univariable analysis, age (OR = 4.207; 95% CI: 2.362–8.798; *p* < 0.001), BMI (OR = 4.049; 95% CI: 1.996–12.114; *p* < 0.001), and serum CD93 (OR = 0.398; 95% CI: 0.054–0.869; *p* = 0.004) were each significantly associated with EC. In the multivariable model, all three variables retained statistical significance. Age remained an independent predictor of EC (OR = 3.345; 95% CI: 1.715–7.498; *p* < 0.001), as did BMI (OR = 3.539; 95% CI: 1.954–11.963; *p* < 0.001). Serum CD93 was inversely associated with EC in the multivariable model (OR = 0.521; 95% CI: 0.061–0.720; *p* < 0.001).

### 3.5. Diagnostic Performance of Serum CD93

The diagnostic value of serum CD93 for discriminating EC from healthy controls, as assessed by ROC curve analysis, is presented in Table 5. The AUC was 0.845 (95% CI: 0.759–0.921). The optimal cut-off value, determined by the Youden index, was 7.338 ng/mL, yielding a sensitivity of 82.6%, specificity of 74.3%, PPV of 80.9%, and NPV of 76.5%. Application of this threshold correctly classified 38 of 46 EC cases and 26 of 35 control subjects, with 8 false-negative and 9 false-positive results.

## 4. Discussion

The present study demonstrates that serum CD93 levels are significantly reduced in patients with EC compared with healthy controls and that this biomarker retains independent diagnostic value after adjustment for established risk factors, including age and BMI [25]. With an AUC of 0.845, a sensitivity of 82.6%, and a specificity of 74.3%, serum CD93 exhibits robust performance characteristics that compare favorably with existing serum biomarkers for endometrial malignancy. To our knowledge, this is one of the very few clinical investigations to evaluate circulating CD93 protein levels in patients with EC, thereby providing clinical validation for computational predictions derived from pan-cancer transcriptomic datasets [22,23].

A substantial body of evidence has established CD93 as a pro-angiogenic molecule that is upregulated in the tumor vasculature of numerous solid malignancies. Langenkamp et al. [16] demonstrated that elevated CD93 expression in glioblastoma blood vessels regulates cytoskeletal rearrangements and correlates with reduced patient survival. Lugano et al. [11] subsequently elucidated the molecular mechanism by which CD93 promotes β1 integrin activation and fibronectin fibrillogenesis during tumor angiogenesis, identifying the CD93–MMRN2 axis as a central mediator of vascular maturation in the TME. This paradigm has been extended to gastric cancer, where Li et al. [18] reported that CD93 serves as a biomarker correlating with immunosuppressive microenvironment features, and to hepatocellular carcinoma, where Jiang et al. [19] showed that CD93 overexpression is associated with immune evasion and unfavorable prognosis. More recently, Yang et al. [20] confirmed the prognostic significance of elevated vascular CD93 in colorectal cancer. In contrast to these findings, our data reveal that serum CD93 is markedly downregulated in EC. This inverse pattern aligns with the observations of Tong et al. [22] and Zhang et al. [23], who, using large-scale pan-cancer transcriptomic analyses of The Cancer Genome Atlas (TCGA) data, identified uterine corpus endometrial carcinoma (UCEC) as one of the few malignancies in which CD93 expression is significantly decreased relative to normal tissue. Our study extends these bioinformatics-based observations to the protein level in the circulation, providing direct clinical evidence that reduced serum CD93 concentrations characterize the presence of endometrial malignancy.

The biological basis for CD93 downregulation in EC requires interpretation in the context of two fundamental aspects of CD93 molecular biology. First, as a transmembrane glycoprotein, CD93 undergoes ectodomain shedding mediated by matrix metalloproteinases (MMPs) and ADAM-family sheddases, releasing sCD93 into the circulation from the surface of endothelial and activated myeloid cells [13]. The shed sCD93 ectodomain retains its CTLD and EGF-like repeat domains, preserving its capacity to bind integrins and facilitate efferocytosis. In the context of EC, several mechanisms may account for the reduced circulating sCD93 levels observed in our cohort. Transcriptional downregulation of CD93 in endometrial tumor tissue, as suggested by TCGA analyses [22,23], would directly reduce the transmembrane CD93 pool available for proteolytic shedding, resulting in diminished sCD93 release into the circulation. Alternatively, dysregulation of sheddase activity—such as altered MMP-2 or ADAM protease expression—within the EC microenvironment could impair ectodomain cleavage independently of CD93 transcription. Furthermore, local sequestration of sCD93 within the tumor stroma through enhanced binding to extracellular matrix integrins may reduce the fraction entering the systemic circulation. Second, following ectodomain shedding, the remaining intracellular domain (ICD) of CD93 undergoes further cleavage and may translocate to the nucleus, where it participates in transcriptional regulation [10]. The primary intracellular signaling mechanism described for CD93 involves the CD93/Cbl/Crk adaptor protein pathway, which regulates Rho family GTPase activity and governs endothelial cytoskeletal dynamics, cell migration, and adhesion [10]. In EC, diminished CD93 surface expression would attenuate CD93/Cbl/Crk-mediated Rho GTPase signaling, potentially impairing endothelial barrier function and altering tumor-associated vascular architecture—a finding consistent with the demonstration by Vemuri et al. [26] that CD93 is essential for maintaining endothelial barrier integrity and limiting metastatic dissemination. Loss of CD93 and its associated Cbl/Crk signaling could therefore lead to dysregulated angiogenesis within the endometrial stroma, contributing to a vascular phenotype permissive of tumor initiation and progression.

Additional mechanistic considerations include the interaction between CD93 and IGFBP7, a negative regulator of angiogenesis and tumor growth [12,27]. Xu et al. [27] resolved the structural basis of the CD93–IGFBP7 interaction, demonstrating that this complex modulates endothelial cell behavior. Coordinated downregulation of the CD93–IGFBP7 axis in the endometrial TME may shift the angiogenic balance toward a more permissive state for tumor development. The unique vascular biology of the endometrium, which undergoes cyclical angiogenesis, breakdown, and regeneration under hormonal regulation, provides a distinctive microenvironmental context in which CD93 loss may disrupt vascular homeostasis and facilitate the transition from endometrial hyperplasia to invasive malignancy [28]. Epigenetic mechanisms, including promoter hypermethylation and microRNA-mediated post-transcriptional silencing, may also contribute to selective CD93 suppression in endometrial tumors, although this hypothesis remains speculative [22,29].

The organ-specificity of CD93 regulation in cancer is increasingly recognized. As Orlandini [30] recently emphasized, the cellular context in which CD93 operates, including the local cytokine milieu, extracellular matrix composition, and immune cell repertoire, fundamentally shapes its functional output. The endometrial microenvironment, characterized by estrogen and progesterone signaling, cyclical tissue remodeling, and a distinctive immune cell composition, may provide a unique context in which CD93 operates as a tumor-suppressive rather than tumor-promoting factor. This notion is further supported by the observation in TCGA analyses that CD93 downregulation in UCEC correlates with favorable immune infiltration patterns [22,23], raising the intriguing possibility that CD93 loss in EC may paradoxically reflect or contribute to immune activation.

The diagnostic performance of serum CD93 observed in our study compares favorably with that of conventional serum biomarkers that have been evaluated for EC. CA-125, the most widely studied circulating marker in gynecological oncology, has demonstrated limited utility for EC screening, with reported sensitivities ranging from 10% to 30% for early-stage disease and 50% to 80% for advanced-stage disease [5,6]. The AUC values for CA-125 in distinguishing EC from benign conditions have ranged between 0.60 and 0.75 in published series [5]. Similarly, CA19-9 and HE4 have shown inconsistent performance across studies and populations [6,7]. In the present cohort, the AUC of 0.845 for serum CD93 substantially exceeds these benchmarks, suggesting superior discriminative ability. Furthermore, the observation that serum CD93 retained independent predictive value after adjustment for age and BMI, two of the most well-established risk factors for EC [2,3], underscores its potential contribution as a biomarker that captures pathobiological processes beyond conventional risk determinants.

Within the EC group, serum CD93 levels did not demonstrate significant associations with histological subtype, tumor grade, tumor size, LVSI, lymph node metastasis, or disease recurrence. However, the borderline association between lower CD93 levels and deeper myometrial invasion is noteworthy. Myometrial invasion depth is a well-recognized prognostic indicator in EC and a key determinant of surgical staging [4,31]. The trend toward lower CD93 concentrations in patients with ≥1/2 myometrial invasion, alongside the lower CD93 levels observed in patients with recurrence, LVSI, and para-aortic lymph node metastasis, raises the possibility that CD93 downregulation may be more pronounced in the context of locally aggressive or advanced disease phenotypes. The absence of statistical significance for these comparisons is likely attributable to the limited sample size and the consequent lack of statistical power, particularly for subgroups with small numbers of events.

### 4.1. Clinical Significance

EC diagnosis currently relies on invasive endometrial sampling, while available serum biomarkers such as CA-125 demonstrate limited sensitivity for early-stage disease. Pan-cancer transcriptomic analyses have identified CD93, an angiogenesis-related transmembrane glycoprotein, as significantly downregulated in uterine corpus endometrial carcinoma; however, no clinical study has previously measured circulating CD93 protein levels in this patient population. Investigating serum CD93 was necessary to determine whether this computationally derived observation translates into a clinically useful circulating biomarker. Demonstrating independent diagnostic performance (AUC 0.845) exceeding published benchmarks for CA-125 supports the potential utility of CD93 in non-invasive risk stratification for suspected endometrial malignancy.

### 4.2. Strengths and Clinical Implications

The present study has several notable strengths. It is one of the few studies to quantify serum CD93 protein levels in patients with EC, thereby translating bioinformatics-derived hypotheses into clinical evidence. From a clinical perspective, the identification of serum CD93 as a non-invasive diagnostic marker for EC has several potential applications. First, it may serve as a triage tool for women presenting with abnormal uterine bleeding, particularly postmenopausal bleeding, to identify those who are most likely to harbor malignancy and therefore warrant prompt tissue sampling. Second, integration of CD93 into multi-marker panels, potentially alongside HE4, CA-125, or molecular classifiers, could enhance diagnostic accuracy for EC [6,7]. Third, the trend toward lower CD93 levels in patients with adverse pathological features suggests that CD93 may ultimately have prognostic relevance, although this requires confirmation in adequately powered studies.

### 4.3. Limitations

Several limitations of this study should be acknowledged. First, the sample size was relatively modest and derived from a single institution, which may limit the generalizability of the findings to broader populations with different ethnic, genetic, and environmental backgrounds. The case–control design carries inherent limitations, including the potential for spectrum bias and overestimation of diagnostic accuracy compared with prospective screening studies employing consecutive patient enrollment; the reported AUC of 0.845 should therefore be regarded as a preliminary estimate requiring external validation. Second, significant differences in age, BMI, and menopausal status between the cancer and control groups are inherent to the epidemiology of EC; although multivariable adjustment was performed, residual confounding—including from unmeasured variables such as diabetes mellitus, exogenous hormone use, statin therapy, and genetic susceptibility—cannot be entirely excluded. Future studies employing frequency-matched or propensity-score-matched cohorts are recommended. Third, CA-125 and CA19-9 were measured only in the cancer group, precluding a direct head-to-head comparison of diagnostic performance between CD93 and these conventional markers within the same study population; indirect comparisons with published AUC values should be interpreted with caution. Fourth, this study assessed only circulating CD93 protein levels; correlation with tissue-level CD93 expression by immunohistochemistry in matched tumor specimens would have provided insight into whether reduced serum levels reflect decreased tissue expression, altered proteolytic shedding kinetics, enhanced local sequestration of sCD93 within the tumor stroma, or a combination of these mechanisms. Fifth, the case–control design precludes evaluation of the temporal dynamics of serum CD93 during disease progression, treatment response, and surveillance. Sixth, the absence of molecular classification data (e.g., POLE mutation status, microsatellite instability, p53 status) for the EC cohort limits our ability to contextualize CD93 levels within the contemporary molecular taxonomy of EC [8,31,32]. CD93 expression may vary across TCGA-defined molecular subtypes, and future validation studies should incorporate molecular classification as a stratification variable. Finally, the small number of non-endometrioid cases (4 serous, 2 clear cell, 1 carcinosarcoma) precluded meaningful subtype-specific subgroup analyses.

### 4.4. Future Directions

Several avenues of investigation emerge from this work. Large-scale, prospective, multicenter studies with demographically matched cohorts are needed to validate serum CD93 as a diagnostic biomarker across diverse populations and to establish robust cut-off values with external validation. Inclusion of patients with endometrial hyperplasia, healthy asymptomatic women without gynecological pathology, and other benign uterine conditions as additional comparator groups would help to define the specificity of CD93 downregulation for invasive malignancy as opposed to benign endometrial proliferative changes and to establish population-level reference ranges. Longitudinal studies tracking serum CD93 levels through the continuum of endometrial carcinogenesis from hyperplasia to early- and advanced-stage cancer could clarify whether CD93 decline is an early event amenable to screening-level detection. A prospective validation study should systematically measure CA-125, CA19-9, and HE4 in both cases and controls to enable direct within-study comparisons and evaluation of multi-marker panels.

At the mechanistic level, functional studies using EC cell lines and in vivo models are warranted to determine whether CD93 downregulation is a passenger phenomenon or a causal contributor to endometrial oncogenesis. Specifically, investigations should explore the interaction between CD93, its ligands MMRN2 and IGFBP7 [11,12,27], and the downstream signaling pathways, including integrin-mediated adhesion, CD93/Cbl/Crk-mediated Rho GTPase activation [10,33], and ectodomain shedding dynamics [13] in the endometrial TME. Paired serum and tissue analyses, encompassing immunohistochemistry and quantitative mRNA expression in tumor and adjacent normal endometrium, are essential to elucidate whether reduced circulating sCD93 levels reflect diminished tissue expression, altered sheddase activity, or local stromal sequestration. Integration of CD93 measurements with TCGA-based molecular classification schemes [8,31,32] and polygenic risk scores derived from GWAS data may reveal subtype-specific patterns of CD93 dysregulation with potential implications for personalized diagnostic and therapeutic strategies, including anti-angiogenic and immunotherapy combinations [21].

## 5. Conclusions

In conclusion, this study provides foundational evidence supporting serum CD93 as a novel, minimally invasive circulating biomarker with promising diagnostic utility for EC. The significant inverse association between circulating CD93 concentrations and EC malignancy, coupled with diagnostic performance metrics exceeding published benchmarks for conventional serum markers, positions serum CD93 as a candidate biomarker warranting further clinical validation. The observation that CD93 downregulation in EC diverges fundamentally from the tumor-promoting, pro-angiogenic functions ascribed to CD93 in other solid malignancies highlights the critical importance of tissue context-specific mechanisms, including ectodomain shedding dynamics and CD93/Cbl/Crk intracellular signaling—in biomarker biology and TME regulation. These findings should be interpreted in light of the study’s limitations, including the absence of paired tissue CD93 expression data, TCGA molecular classification, and the constraints of a single-center case–control design, and require confirmation in large-scale, prospective, multicenter cohorts before clinical translation can be recommended.

## Figures and Tables

**Table 1:** Baseline characteristics of endometrial cancer and control groups.

Variable	Endometrial Cancer (*n* = 46)	Control (*n* = 35)	*p*-Value
Age (years), median [IQR]	63.00 [55.75–69.00]	45.00 [32.50–55.00]	<0.001
BMI (kg/m^2^), median [IQR]	37.00 [30.00–43.00]	28.00 [27.00–29.00]	<0.001
Parity, median [IQR]	3.00 [2.00–3.00]	2.00 [1.00–3.00]	0.377
Menopausal status, *n* (%)			<0.001
Premenopausal	5 (11.1)	21 (60.0)	
Postmenopausal	40 (88.9)	14 (40.0)	

Data are presented as median [interquartile range] or *n* (%). BMI, body mass index; IQR, interquartile range.

**Table 2:** Biomarker levels in the endometrial cancer and control groups.

Biomarker	Endometrial Cancer (*n* = 46)	Control (*n* = 35)	*p*-Value
Serum CD93 (ng/mL), median [IQR]	4.55 [3.51–6.97]	10.24 [7.18–12.14]	<0.001
CA-125 (U/mL), median [IQR]	15.9 [7.6–26.8] (*n* = 42)		
CA19-9 (U/mL), median [IQR]	13.0 [6.9–19.0] (*n* = 41)		

Data are presented as median [interquartile range]. IQR, interquartile range. CA-125 and CA19-9 were not measured in the control group.

**Table 3:** Association Between Serum CD93 Levels and Clinicopathological Parameters in Endometrial Cancer.

Variable	*n* (%)	CD93 (ng/mL), Median [IQR]	*p*-Value
Menopausal status			0.396
Premenopausal	5 (11.1)	7.75 [3.50–12.09]	
Postmenopausal	40 (88.9)	4.48 [3.48–6.79]	
Histological type			0.706
Endometrioid carcinoma	34 (73.9)	4.05 [3.30–6.77]	
Serous carcinoma	4 (8.7)	6.06 [5.37–6.30]	
Clear cell carcinoma	2 (4.3)	4.60 [3.52–5.69]	
Carcinosarcoma	1 (2.2)	4.50 [4.50–4.50]	
Grade			0.722
Grade 1	16 (34.8)	3.86 [3.28–5.20]	
Grade 2	11 (23.9)	4.46 [3.06–5.37]	
Grade 3	10 (21.7)	5.19 [3.53–6.97]	
Tumor size (≤4 cm vs. >4 cm)			0.506
≤4 cm	15 (32.6)	3.64 [2.66–6.12]	
>4 cm	22 (47.8)	4.55 [3.65–6.71]	
Myometrial invasion			0.093
<1/2	30 (65.2)	4.68 [3.57–6.83]	
≥1/2	11 (23.9)	3.52 [2.82–4.71]	
LVSI			0.275
Absent	28 (60.9)	4.37 [3.55–6.83]	
Present	11 (23.9)	3.52 [2.82–5.49]	
Pelvic LN metastasis			1.000
Absent	36 (78.3)	4.35 [3.33–6.77]	
Present	4 (8.7)	4.18 [3.35–6.01]	
Para-aortic LN metastasis			0.457
Absent	36 (78.3)	4.48 [3.33–6.79]	
Present	4 (8.7)	3.88 [3.35–4.38]	
Recurrence			0.218
Absent	37 (80.4)	4.46 [3.50–6.77]	
Present	3 (6.5)	2.83 [2.82–3.66]	

Data are presented as median [interquartile range]. LVSI, lymphovascular space invasion; LN, lymph node; IQR, interquartile range.

**Table 4:** Logistic Regression Analysis for Endometrial Cancer.

Variable	Univariable OR	95% CI	*p*-Value	Multivariable OR	95% CI	*p*-Value
Age (years)	4.207	2.362–8.798	<0.001	3.345	1.715–7.498	<0.001
BMI (kg/m^2^)	4.049	1.996–12.114	<0.001	3.539	1.954–11.963	<0.001
CD93 (ng/mL)	0.398	0.054–0.869	0.004	0.521	0.061–0.720	<0.001

L2-regularized logistic regression was applied using the scikit-learn framework (C = 1.0). Bootstrap-based 95% confidence intervals were estimated from 1000 iterations. OR, odds ratio; CI, confidence interval; BMI, body mass index.

**Table 5:** Diagnostic value of serum CD93 for Endometrial cancer.

Parameter	Value	
AUC	0.845	
95% CI (AUC)	0.759–0.921	
Optimal cut-off (ng/mL)	7.338	
Sensitivity (%)	82.6	
Specificity (%)	74.3	
PPV (%)	80.9	
NPV (%)	76.5	
	Predicted Cancer	Predicted Control
Actual Cancer	38	8
Actual Control	9	26

AUC, area under the curve; CI, confidence interval; PPV, positive predictive value; NPV, negative predictive value.

## Data Availability

The datasets generated and/or analyzed in the current study are available from the corresponding author upon reasonable request. All data supporting the findings of this study are included within the article.
